# Docosahexaenoic Acid Promotes Cd Excretion by Restoring the Abundance of *Parabacteroides* in Cd-Exposed Mice

**DOI:** 10.3390/molecules28104217

**Published:** 2023-05-21

**Authors:** Jianzhen Liao, Siyuan Bi, Zhijia Fang, Qi Deng, Yinyan Chen, Lijun Sun, Yongqing Jiang, Linru Huang, Ravi Gooneratne

**Affiliations:** 1 College of Food Science and Technology, Guangdong Provincial Key Laboratory of Aquatic Product Processing and Safety, Guangdong Provincial Engineering Technology, Research Center of Marine Food, Key Laboratory of Advanced Processing of Aquatic Products of Guangdong Higher Education Institution, Guangdong Ocean University, Zhanjiang 524088, China; liaojianzhen604@163.com (J.L.); bisiyuan0129@126.com (S.B.); dengqi@gdou.edu.cn (Q.D.); rqxchenyinyan@163.com (Y.C.); suncamt@126.com (L.S.); linruhuang7@163.com (L.H.); 2 Shenzhen Jinyue Test Technology Co., Ltd., Shenzhen 510100, China; 3 Shenzhen Lvshiyuan Biotechnology Co., Ltd., Shenzhen 510100, China; 4 Department of Wine, Food and Molecular Biosciences, Lincoln University, Lincoln 7647, New Zealand; ravi.gooneratne@lincoln.ac.nz

**Keywords:** cadmium, gut microbiota, docosahexaenoic acid, cadmium excretion, *Parabacteroides distasonis*, succinic acid

## Abstract

As a common harmful pollutant, cadmium (Cd) can easily enter the human body through the food chain, posing a major threat to human health. Gut microbiota play a key role in Cd absorption. Docosahexaenoic acid (DHA) is thought to have a potential role in the treatment of Cd poisoning. This study investigated the therapeutic effect and mechanism of DHA in Cd-exposed mice from the perspective of the gut microbiota. The results showed that DHA significantly increased the Cd content in feces and decreased the Cd accumulation in the organs of mice. The gut microbiota results showed that DHA significantly restored the abundance of Parabacteroides in the gut microbiota of Cd-exposed mice. *Parabacteroides distasonis* (*P. distasonis*), a representative strain of the *Parabacteroides*, also showed Cd- and toxicity-reduction capabilities. *P. distasonis* significantly restored the gut damage caused by Cd exposure. At the same time, *P. distasonis* reduced the Cd content in the liver, spleen, lung, kidneys, gut, and blood to varying degrees and significantly increased the Cd content in feces. The succinic acid produced by *P. distasonis* plays an important role in promoting Cd excretion in Cd-exposed mice. Therefore, these results suggest that *P. distasonis* may have a potential role in DHA-mediated Cd excretion in Cd-exposed mice.

## 1. Introduction

In 2023, about 15.4% of the Chinese population was at risk of high exposure to Cd through their diet [1]. Cd contamination in food has been a chronic problem [2]. Cd is excreted slowly after entering the body. Most of the accumulated Cd is first absorbed in the intestine and then transported to other organs [3,4], mainly accumulating in the liver [5], kidneys [6], and intestine [7], resulting in varying degrees of damage to the organs [8]. Cd exposure can affect the diversity and abundance of gut microbiota and cause significant changes in the metabolic function of gut microbiota [9]. Identifying a means of effectively controlling the damage from Cd has been a puzzle for researchers.

ω-3 unsaturated fatty acids may help to prevent neurodegenerative diseases caused by Cd exposure [10]. However, the human body cannot synthesize ω-3 and ω-6 unsaturated fatty acids, so they must be obtained from food [11]. DHA and EPA mixtures were reported to protect human hepatocellular cells (Hep G2) from acute Cd exposure (24 or 48 h) [12]. In addition, fish oil rich in unsaturated fatty acids can regulate gut microbiota [13,14]. Our previous studies have shown that adding the unsaturated fatty acid oleic acid (OA) can reduce Cd accumulation, promote Cd excretion, and significantly restore the diversity of the gut microbiota, confirming the ability of OA to reduce Cd [15]. Therefore, unsaturated fatty acids can potentially treat Cd poisoning and regulate the gut microbiota.

Studies have shown that the gut microbiota is a key determinant of host health [16,17]. As a part of the intestinal barrier, the gut microbiota is closely related to gut environmental homeostasis, and an imbalance in the gut microbiota may lead to damage to the gut defense system [18,19]. The presence of symbiotic bacteria in the gut provides the first barrier to toxins [20]. Cd exposure can increase the relative abundance of Cd-resistant microorganisms in the gut microbiota [21]. Studies have confirmed that gut microbiota can solidify and absorb some heavy metals, thus reducing their distribution in human tissues [22]. Gut microbiota can remove metals and detoxify and eliminate toxic compounds in the body by improving gut microbial balance and excretion [23]. Studies have shown that Cd exposure disrupts the gut microbiota in mice, leading to increased gut permeability [24] and a reduced abundance of probiotics such as *Lactobacillus* and *Bifidobacteria* [25,26]. Some gut probiotics also play an essential role in promoting Cd excretion [15,27]. For example, in mouse models, *Lactobacillus plantarum* CCFM8610 can reduce Cd accumulation in organs by increasing Cd levels in feces [28,29]. Hence, the gut microbiota has the potential to mitigate Cd-induced harm.

This study aimed to elucidate the protective effect of the unsaturated fatty acid DHA on Cd-exposed mice from the perspective of the gut microbiota and to identify key gut microorganisms that promote Cd excretion. The mechanism of Cd reduction and the emission promotion of key bacterial groups was analyzed. Overall, this study may provide a safe and effective way to promote Cd excretion through dietary supplementation.

## 2. Results

### 2.1. Effects of DHA on Cd Levels in Cd-Exposed Mice

**Figure 1:**
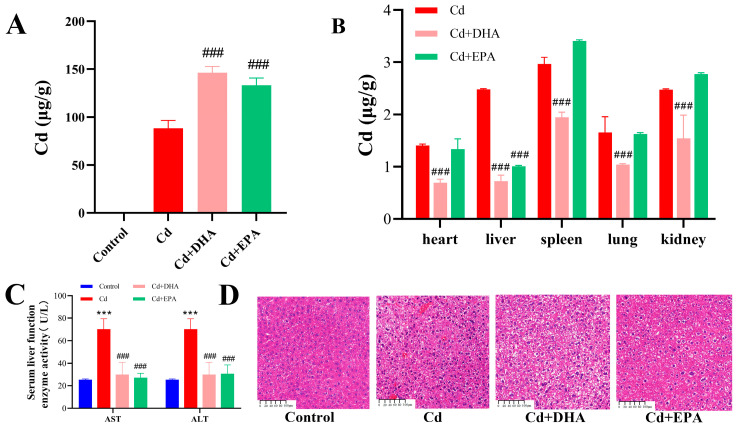
Effect of DHA on Cd content in Cd-exposed mice. (**A**) Cd content in feces. (**B**) Cd content in organs. (**C**) Serum AST and ALT activity levels. (**D**) Liver section. Magnification 10×; the reference line is 100 μm. *** *p* < 0.001 represented a significant difference compared with the control group. ^###^ *p* < 0.001 represented a significant difference compared with the Cd group.

To investigate the ability of the unsaturated fatty acids DHA and EPA to affect Cd excretion in mice, as shown in Figure 1A, the Cd content in mouse feces was determined. Compared with the Cd group, the unsaturated fatty acids DHA and EPA significantly increased the fecal Cd content (*p* < 0.001), and the Cd concentration in the feces of mice in the DHA treatment group increased by 65.8%. The results show that DHA had a strong ability to promote Cd excretion in mouse feces. On the other hand, Cd levels in the heart, liver, spleen, lung, and kidneys were significantly decreased after oral DHA administration (*p* < 0.001), while only liver Cd levels were reduced in the EPA treatment group (Figure 1B). In addition, the activities of serum AST and ALT in Cd-exposed mice were decreased after DHA and EPA treatment (*p* < 0.001), and the integrity of the liver tissue was restored (Figure 1C,D). These results suggest that oral DHA can reduce Cd accumulation in organs and promote fecal Cd excretion in mice.

### 2.2. Effects of DHA on Gut Microbiota of Cd-Exposed Mice

**Figure 2:**
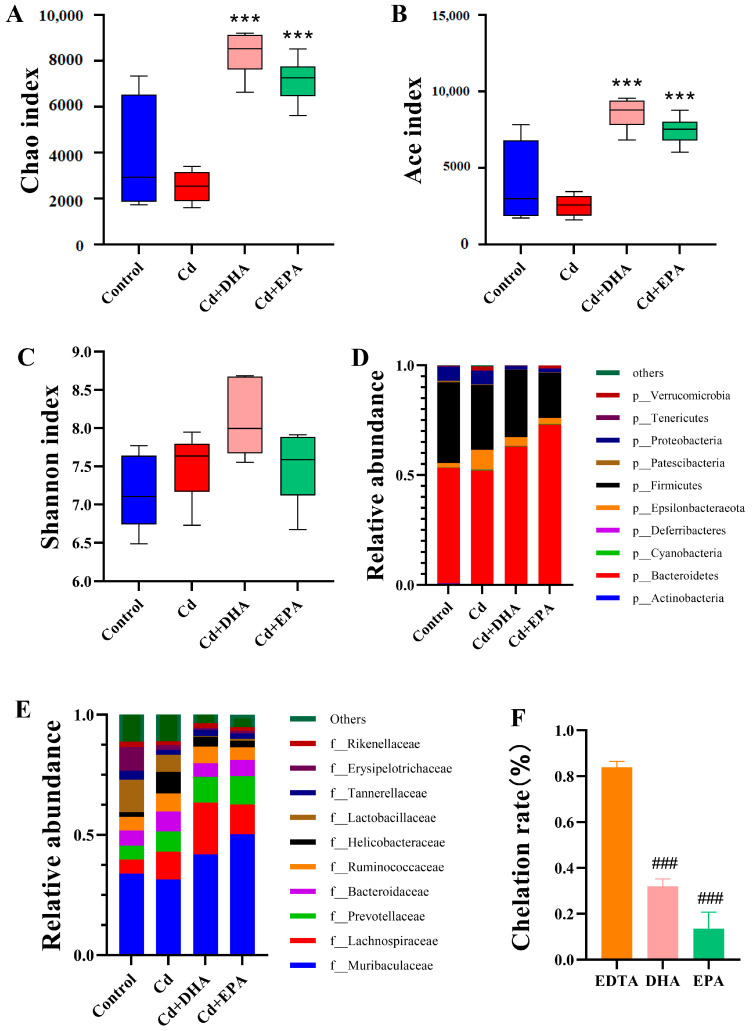
Analysis of the ability of DHA to reverse Cd-induced changes in gut microbiota and cause Cd chelation in mice. (**A**) Chao1 index. (**B**) Ace index. (**C**) Shannon index. (**D**) Relative abundance of top 10 gut microbiota at the phylum level. (**E**) Relative abundance of top 10 gut microbiota at the family level. (**F**) Determination of Cd-chelating properties of DHA and EPA via Cd-CAS liquid assay. *** *p* < 0.001 represented a significant difference compared with the Cd group. ### *p* < 0.001 represented a significant difference compared with the EDTA group.

The effects of the unsaturated fatty acids DHA and EPA on gut microbiota were analyzed using high-throughput 16S rDNA sequencing. The higher the alpha diversity index of the gut microbiota, the better the richness and diversity of bacterial species and the more stable the gut microbiota. After the exposure to Cd, the alpha diversity indexes Chao1 and Ace showed a decreasing trend (Figure 2A,B). Compared to the Cd group, the addition of DHA and EPA significantly restored both indexes (*p* < 0.001). However, the Shannon index showed no significant difference between the groups (Figure 2C).

Next, we analyzed the relative abundance of gut microbiota at the phylum level. As shown in Figure 2D, the relative abundances of *Bacteroidetes* and *Firmicutes* decreased in the Cd group compared to the control group. After DHA treatment, the abundances of *Bacteroidetes* and *Firmicutes* were restored. At the family level, the abundances of *Muribaculaceae*, *Lactobacillaceae*, *Tannerellaceae*, and *Erysipelotrichaceae* decreased after Cd exposure. However, after DHA treatment, the changes in gut microbiota such as *Muribaculaceae*, *Lactobacillaceae*, and *Bacteroidaceae* were significantly ameliorated (Figure 2E). These results showed that DHA significantly reversed Cd’s inhibition of the abundance of gut microbiota, especially *Bacteroidetes*. We also evaluated the Cd-chelating properties of DHA and EPA. As shown in Figure 2F, the Cd chelation rate of EDTA is significantly higher than that of DHA and EPA, indicating that EDTA has a strong Cd-chelating capacity. Compared with the EPA-treated group, DHA exhibited a greater Cd-chelating capacity and thus induced higher fecal Cd excretion (Figure 1A and Figure 2F).

### 2.3. DHA Restored the Abundance of Parabacteroides in Cd-Exposed Mice

**Figure 3:**
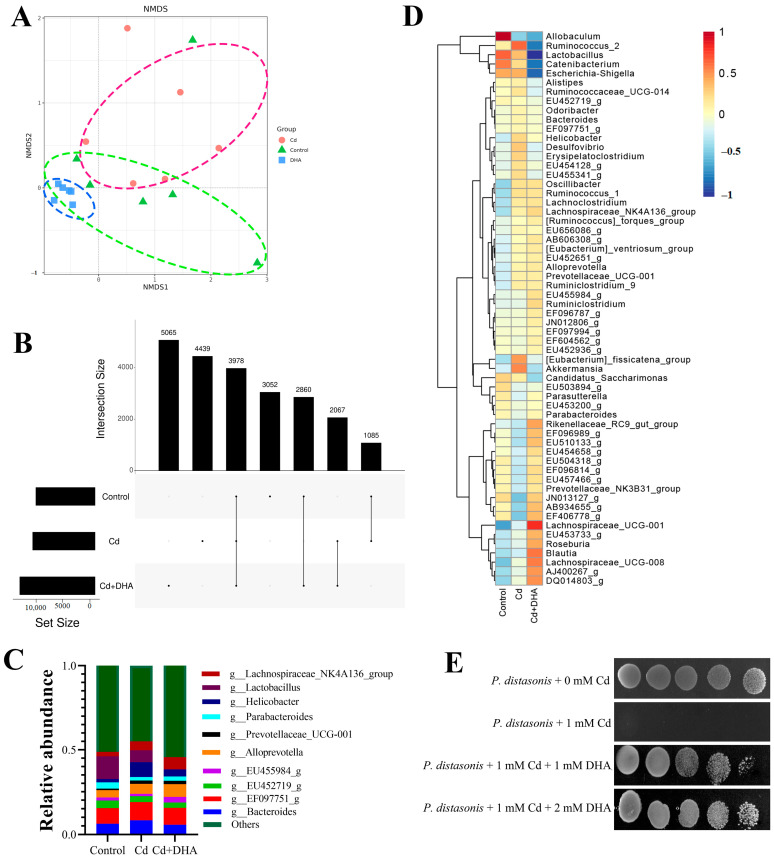
Effect of DHA on gut microbiota of Cd-exposed mice. (**A**) NMDS map of beta diversity of gut microbiota; (**B**) 97% similarity in the Upset diagrams of the OTUs in the control group, Cd group, and DHA-processing group; (**C**) the relative abundances of the top 10 gut microbiota at the genus level; (**D**) heatmap of gut microbiota abundance at the genus level; (**E**) sensitivities of *P. distasonis* to Cd evaluated: *P. distasonis* were spotted onto MRS medium.

The NMDS diagram for the beta diversity analysis shows that the overlap between the DHA treatment and control groups is greater than the overlap between the Cd-treated and control groups (Figure 3A). When comparing the number of OTUs shared by the gut microbiota at 97% of the same level in the UpSet diagram (Figure 3B), the number of OTUs shared between the DHA treatment group and the control group (2860) was higher than the number of OTUs shared between the Cd treatment group and the control group (1085). The results of both the NMDS and UpSet maps showed that the structure of the gut microbiota of the DHA treatment group was closer to that of the control group, indicating that DHA has an anti-Cd-toxicity effect on the gut microbiota. At the genus level, the relative abundances of *Parabacteroides* and *Lactobacillus* were lower after Cd exposure than in the control group. However, *Parabacteroides* recovered after treatment with DHA (Figure 3C). Additionally, a heatmap analysis of the gut microbiota composition of the first 10 microorganisms at the genus level showed that *Parabacteroides* recovered significantly after DHA supplementation in Cd-exposed mice (Figure 3D). These results suggest that the reduction in Cd content in Cd-exposed mice treated with DHA may be related to *Parabacteroides* in the gut.

*P. distasonis* was one of the representative strains of *Parabacteroides*. Next, we investigated in vitro whether DHA can protect *P. distasonis* against Cd exposure. *P. distasonis* was dripped onto MRS plates with/without 1 mM Cd. The results showed the protective effect of DHA by treatment with 1 mM DHA. When *P. distasonis* was treated with 1 mM DHA, Cd resistance of DHA was observed (Figure 3E).

### 2.4. Effects of P. distasonis on Gut Tissues of Cd-Exposed Mice

**Figure 4:**
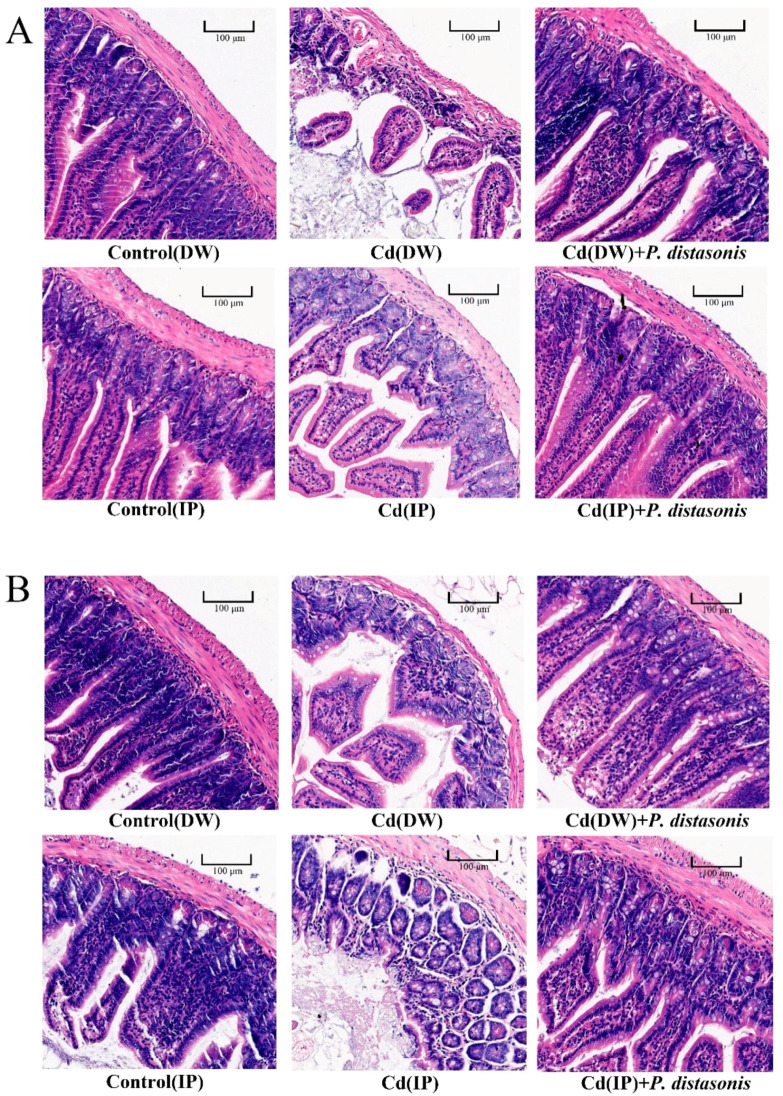
Effect of *P. distasonis* on the gut histopathological sections of Cd-exposed mice. (**A**) H&E-stained sections of mouse jejunum. (**B**) H&E-stained sections of mouse ileal tissue. The magnification is 20×, and the reference line is 100 μm.

To investigate whether the protective effect of *P. distasonis* in Cd-exposed mice was consistent with the effect seen in in vitro studies, pathological sections of jejunum and ileum tissues from Cd-exposed mice were observed. As shown in Figure 4, the sections of the jejunum and ileum in the control group had normal morphologies, intact gut walls, and regular villi. Compared with the control group, the damage to the gut in the Cd group was more severe, including shedding villi, obvious wear, and the thinning of the gut wall. Compared to the Cd group, these phenomena were alleviated after treatment with *P. distasonis*. These results suggest that *P. distasonis* has a protective effect on the integrity of the gut barrier.

### 2.5. Effects of P. distasonis on Cd Content in Cd-Exposed Mice

**Figure 5:**
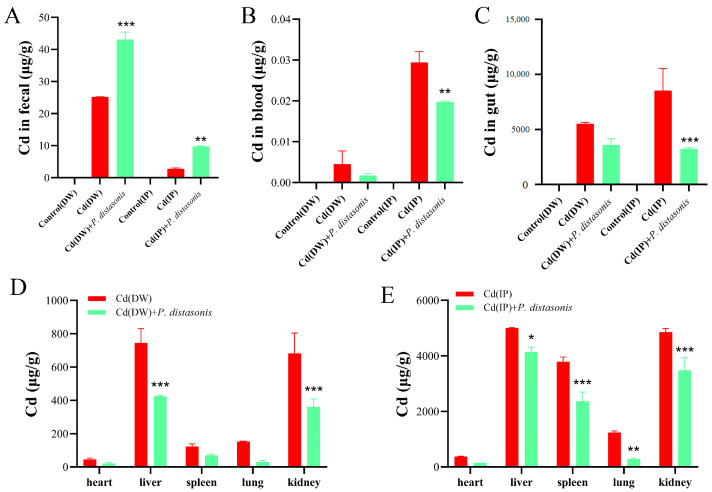
Effects of *P. distasonis* on Cd levels in Cd-exposed mice. (**A**) Cd content in feces. (**B**) Blood Cd content. (**C**) Gut Cd content. (**D**) Cd content in heart, liver, spleen, lung and kidneys of DW group. (**E**) Cd content in the heart, liver, spleen, lung and kidneys of IP group. * *p* < 0.05, ** *p* < 0.01, *** *p* < 0.001 represented a significant difference compared with the Cd group.

To assess the effect of *P. distasonis* on Cd excretion in Cd-exposed mice, Cd levels were determined in the feces, organs, and blood. Compared to the Cd group, the fecal Cd content of mice treated with *P. distasonis* was significantly increased (*p* < 0.001) (Figure 5A). The results indicate that *P. distasonis* could greatly promote Cd excretion in the feces of Cd-exposed mice. In the IP group, Cd contents in the blood and organs of the mice (except the heart) were inhibited to varying degrees after the addition of *P. distasonis* (*p* < 0.05). Cd levels in the liver and kidneys were significantly decreased in the DW group (*p* < 0.001), and no other significant differences were found (Figure 5B–E). These results indicate that *P. distasonis* can induce Cd excretion in the feces of Cd-exposed mice by reducing the contents of Cd in the blood and organs, thus reducing the absorption of Cd by the body.

### 2.6. Effect of Succinic Acid on Cd Content in Cd-Exposed Mice

**Figure 6:**
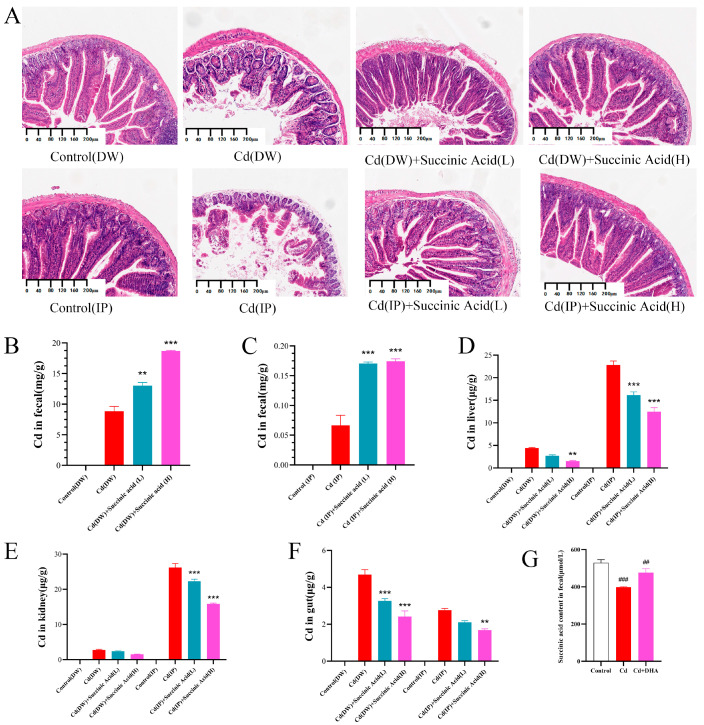
Effect of succinic acid on Cd level in Cd-exposed mice. (**A**) H&E-stained section of gut tissue. (**B**) Cd content in feces of DW group. (**C**) Fecal Cd content in IP group. (**D**) Cd content in liver. (**E**) Cd content in kidney. (**F**) Gut Cd content. (**G**) Succinic acid content in feces of DW group. ** *p* < 0.01, *** *p* < 0.001 represented a significant difference compared with the Cd group. ^##^ *p* < 0.01, ^###^ *p* < 0.001 represented a significant difference compared with the control group.

To further investigate whether the effects of *P. distasonis* in promoting Cd excretion and alleviating Cd poisoning in Cd-exposed mice were related to its high yield of succinic acid, we first observed pathological sections of gut tissues from Cd-exposed mice. As shown in Figure 6A, the gut villi of the mice were intact without Cd treatment, but after exposure to Cd, the gut villi of the mice were severely damaged. After the addition of succinic acid, the gut damage in the mice was reversed. The results showed that succinic acid could alleviate gut damage in mice exposed to Cd.

To determine the role of succinic acid in Cd excretion, Cd contents in the feces and organs of Cd-exposed mice were further measured. As shown in Figure 6B,C, Cd contents in the feces of mice were significantly increased after treatment with succinic acid (*p* < 0.01). As can be seen from Figure 6D–F, succinic acid has a good reducing effect on Cd contents in organs. According to DW, high doses of succinic acid can reduce Cd contents in both the liver and gut, but there is no significant difference in the kidney. It can be seen from the IP groups that succinic acid could reduce Cd contents in all the organs in this study to varying degrees. Therefore, the addition of succinic acid can promote Cd excretion in Cd-exposed mice, mainly by decreasing Cd contents in organs and increasing Cd contents in feces.

To further investigate whether the succinic acid content in the feces changed before and after treatment with Cd or DHA, the content of succinic acid in the feces of Cd-exposed mice was also measured. As shown in Figure 6G, the succinic acid content in the feces of mice decreased significantly after treatment with Cd. However, compared to the Cd group, the succinic acid content in the feces was significantly restored after the addition of DHA. These results further suggest that DHA may promote Cd excretion by restoring the abundance of *P. distasonis* and its secreted succinic acid.

## 3. Discussion

The question of how to effectively remove Cd from the body has been a trending research topic worldwide. DHA has been reported to increase resistance to Cd [30]. Cd exposure can lead to disorders of the gut microbiota, and studies have shown that the gut microbiota plays an essential role in Cd reduction. Our study found that the unsaturated fatty acid DHA can increase the abundance of *Parabacteroides* in the gut, thereby reducing Cd accumulation and promoting Cd excretion.

### 3.1. Unsaturated Fatty Acid DHA Can Significantly Reduce Cd and Toxicity

As essential dietary supplements in the body, unsaturated fatty acids play an essential role in preventing and alleviating Cd toxicity [31,32]. It has been reported that ω-3 unsaturated fatty acids can alleviate liver toxicity in rats exposed to Cd [33]. The unsaturated fatty acid OA may promote Cd excretion in feces and reduce Cd accumulation in organs [15]. In addition, Cd exposure can reduce the contents of unsaturated fatty acids (such as OA, DHA, and LA) in the muscle and skin of rainbow trout [34]. DHA may protect the liver cells of rainbow trout and improve the resistance of cells to Cd [30]. In our study, DHA showed significant Cd reduction and detoxification benefits, increasing fecal Cd output and significantly reducing Cd levels in the heart, liver, spleen, lungs, and kidneys. This result is consistent with previous reports that fish fed diets rich in DHA have the detoxifying ability to reduce Cd accumulation in their organs [35]. We also found that DHA can effectively reduce the activities of serum AST and ALT and restore the integrity of liver tissue. Cd is transported through the blood to various organs, and liver damage caused by Cd leads to increased membrane fluidity, causing enzymes in the liver to leak into the blood [36]. Therefore, serum AST and ALT are important markers of hepatocyte injury [37,38]. Previous studies have shown that feeding abalone with DHA can promote growth [39]. Liao et al. [40] pointed out that Cd exposure significantly reduced DHA concentrations in the brain of a silver pomfret, but the silver pomfret could biosynthesize DHA from endogenous EPA, thus enhancing its tolerance to Cd. DHA has a protective effect against tissue damage in mice exposed to Cd [41]. Therefore, this study demonstrates the efficacy of DHA in detoxifying Cd, reducing Cd accumulation in organs and promoting Cd excretion in the feces.

### 3.2. The Cd Reduction and Detoxification Effect of DHA Is Closely Related to Its Regulation of Gut Microbiota, Especially Parabacteroides in the Gut

Cd exposure can disrupt the homeostasis of the gut microbiota in rats, and the gut microbiota can mediate the organ toxicity of Cd by activating the immune response, suggesting that the gut microbiota may be a novel target for the treatment of Cd poisoning [42]. In our study, DHA treatment significantly restored the underlying mechanism through which the abundance of gut microbiota in Cd-exposed mice suggests effective Cd excretion. According to the analyses of alpha and beta diversity and OTUs, DHA restored homeostasis to gut microbiota disrupted by Cd exposure. Moreover, DHA restored the relative abundance of the gut microbiota at the phylum and family levels. This is similar to the report by Fang et al. [15]. It has been reported that fish oil rich in DHA can influence the diversity and abundance of the gut microbiota [13,14]. DHA also increased the numbers of potentially beneficial *Lactobacillus* and *Bifidobacteria* in the guts of mice fed a high-fat diet [43,44]. A further analysis of the relative abundance and a heat map at the genus level showed that DHA could significantly restore the abundance of *Parabacteroides*, suggesting that *Parabacteroides* may be the key to reducing Cd toxicity. *Parabacteroides* showed multiple resistances to mercury, zinc, cobalt, and Cd [45]. Studies have shown that dietary licorice enriched with gut *P. distasonis* effectively reduced Cd in the blood of mice [46].

The gut microbiota can play an important role in the degradation of toxins and participate in maintaining the health of the host at different life stages [17,47]. Gut bacteria, as a major component of the gut microbiota, have been shown in many studies to mitigate the damage caused by Cd toxicity. A recent report showed that seven probiotics showed significant protective effects against Cd toxicity in preclinical studies [48]. *Lactobacillus plantarum* CCFM8610 has been reported to promote fecal Cd excretion in mice [29]. After the oral administration of *Lactobacillus plantarum*, the Cd contents in the blood of volunteers were significantly reduced [49]. Yogurt with *Lactobacillus rhamnosus* GR-1 may reduce Cd levels in children and pregnant women [50]. In this study, it was found that the oral administration of *P. distasonis* significantly improved the effect of Cd excretion in Cd-exposed mice, and similar results were observed in our study. The oral administration of *P. distasonis* could alleviate Cd-induced gut damage, reduce Cd levels in the blood and organs (especially the liver, kidneys, and gut), and increase Cd content. *P. distasonis* is the reference strain of *Parabacteroides* [51]. We treated *P. distasonis* with DHA under Cd stress and found that it showed greater tolerance to Cd, further confirming the protective effect of DHA on *P. distasonis.*

### 3.3. Succinic Acid, the Main Product of P. distasonis, Has Good Cd Reduction and Detoxification Effects

Succinic acid has been reported to be the major product of glycolysis in *P. distasonis* [52]. Our study shows that succinic acid can promote Cd excretion in the feces of Cd-exposed mice and reduce Cd accumulation in the liver, kidneys, and gut. This may be because the carboxyl group (-COOH) in succinic acid can undergo complex reactions with Cd, thus achieving the purpose of Cd removal [53,54]. In addition, as an organic acid, succinic acid can inhibit Cd toxicity in plants [55,56] and significantly repair soil Cd contamination [57]. At present, studies on the toxicity of succinic acid to Cd mainly focus on the levels of microorganisms [58,59] and plants [60,61].

Succinic acid also has detoxifying effects on other heavy metals and pollutants. Exogenous succinic acid alleviates Pb-induced oxidative damage and improves the tolerance of *Larix olgensis* seedlings to Pb stress [62]. Sugarcane bagasse treated with succinic acid can be a promising adsorbent for the removal of Cr [63]. Plant root exudates succinic acid can significantly degrade polycyclic aromatic hydrocarbons polluting soil [64].

## 4. Materials and Methods

### 4.1. Reagents and Animals

Cadmium chloride (CdCl_2_, 98%) was purchased from Chengdu Huaxia Chemical Reagent Company (Chengdu, China). Chromeazurol S (CAS, 98%), 2,2′-Bipyridyl (dipy, 98%), Hexadecylpyridinium bromide (HDPB, 98%), and docosahexaenoic acid (DHA, 80%) were purchased from Shanghai Jizhi Biochemical Technology Co., Ltd. (Shanghai, China). Eicosapentaenoic acid (EPA, 65%) was obtained from Tixiae Chemical Industrial Development Co., Ltd. (Shanghai, China). The succinic acid ELISA and assay kit (SY-M03137) was purchased from Shanghai Shuangying Biotechnology Co., Ltd. (Shanghai, China). *P. distasonis* (GDMCC 1.1564) was purchased from Guangdong Provincial Microbial Species Preservation Center.

A total of 108 specific-pathogen-free (SPF) male mice (8 weeks of age) were purchased from SiPeiFu (Beijing, China) Biotechnology Co., Ltd. (SCXK2019-0010). All mice were fed standard commercial rat feed at a temperature control of 25 ± 1 °C and kept in cages with free access to food and water during a 12 h light/dark cycle. This experiment was approved by the Experimental Animal Ethics Committee of Guangdong Ocean University (approval number: GDOU-LAE-2021-020).

### 4.2. DHA and EPA Treatment in Cd-Exposed Mice

Twenty-four SPF mice were randomly divided into four groups (Table 1). In the control group, the mice received normal drinking water without Cd. In the Cd exposure group, the mice were provided drinking water containing 100 μmol/L CdCl_2_. In the DHA and EPA treatment groups, the mice were provided 100 μmol/L CdCl_2_ drinking water and 40 μmol/g/d DHA and EPA orally. The treatment lasted for seven days.

### 4.3. P. distasonis Treatment in Cd-Exposed Mice

Thirty-six SPF mice were randomly divided into six groups (Table 2). The dosages of Cd and *P. distasonis* were consistent with Qixiao et al. [28]. A drinking water control group (DW) was provided water. In the Cd (DW) group, the mice were provided drinking water containing 100 μmol/L CdCl_2_. In the Cd (DW) + *P. distasonis* group, the mice were provided 100 μmol/L CdCl_2_ in their drinking water and 1 × 10^9^ CFU via the oral administration of *P. distasonis*. In the control group, normal saline water was injected intraperitoneally. In the Cd (IP) group, the mice were intraperitoneally injected with 9 μg CdCl_2_. The mice in the Cd (IP) + *P. distasonis* group were intraperitoneally injected with 9 μg CdCl_2_ andwere orally administered 1 × 10^9^ CFU *P. distasonis*. All treatment groups were treated for 7 days.

### 4.4. Succinic acid Treatment in Cd-Exposed Mice

Forty-eight SPF mice were randomly divided into eight groups (Table 3). In the Cd (DW) group, the mice were provided drinking water containing 100 μmol/L CdCl_2_. In the Cd (DW) + Succinic Acid (L) group, the mice were provided 100 μmol/L CdCl_2_ in water and low-dose succinic acid was provided via intragastric administration. In the Cd (DW) + Succinic Acid (H) group, the mice were provided 100 μmol/L CdCl_2_ in water and a high dose of succinic acid (twice the low dose) via gavage. In the Cd (IP) group, the mice were intraperitoneally injected with 9 μg CdCl_2_. In the Cd (IP) + Succinic Acid (L) group, the mice were intraperitoneally injected with 9 μg CdCl_2_ and then received an intragastric administration of low-dose succinic acid. In the Cd (IP) + Succinic Acid (H) group, 9 μg CdCl_2_ was intraperitoneally injected into the mice, and high-dose succinic acid was administered intragastrically. All treatment groups were treated continuously for 21 days.

### 4.5. Determination of Cd Content

Feces samples from each group of mice were collected on time every day to determine the Cd content in the mouse feces. The collected samples (heart, liver, spleen, lung, kidneys, gut, and blood) were used to determine the Cd contents in the organs and blood. The collected samples were uniformly ground and digested with nitric acid (HNO_3_) in the microwave digestion system (GDANA D-360, Guangzhou, China). Finally, the Cd content in the samples was determined using an inductively coupled plasma mass spectrometer (ICP-MS, ThermoFisher, ICAPQ, Bremen, Germany) [65] (*n* = 3).

### 4.6. Determination of Enzyme Activity in Mouse Serum Liver Function

The mouse blood was bathed in a water bath at 37 °C for 30 min and then centrifuged at 4 °C for 20 min at 8000 rpm/min. The serum was collected and measured for liver enzyme activities. The mouse serum ALT and AST kits were used to determine the absorbance at 505 nm (*n* = 3).

### 4.7. Histopathological Observation of Livers and Gut Tracts in Mice

Appropriate amounts of mouse livers and gut tissues were taken and fixed with 4% paraformaldehyde at 4 °C for 24 h. The tissue was embedded in paraffin and sliced to a thickness of 5 μm. It was then stained with hematoxylin and eosin (H&E) slices [66]. Finally, the tissue sections were observed using fluorescence microscopy. At least three images were taken for each processing group.

### 4.8. Sequencing of 16S rDNA Gene in Mouse Gut Microbiota

On the seventh experimental day, the abdomens of the mice were gently massaged several times to promote defecation. Fresh mouse feces were collected and determined according to the method of Fang et al. [15].

### 4.9. Determination of Cd Chelating Ability of DHA and EPA

This experiment was slightly modified according to the previous methods [67]. The initial complex contained: CAS (5 × 10^−3^ mol/L):dipy (0.1 mol/L):HDPB (1 × 10^−3^ mol/L) = 4 mL:1 mL:1 mL. Then, 0.1 mol/L NaOH was added to adjust the complex to green. Five minutes later, 5 mL of a sodium borate–sodium hydroxide buffer solution (pH = 11) was added, and distilled water was used to set the volume to 25 mL. After complete mixing, the change in optical density was measured at 602 nm using an automatic multifunctional enzyme marker (Thermo Fisher, Waltham, MA, USA) within 40 min (*n* = 3). The chelation rate was calculated as described in Li [68].

### 4.10. P. distasonis Analysis of Sensitivity Differences

DHA and *P. distasonis* were cultured in MRS Medium at 37 °C for 48 h to evaluate the protective effect of DHA in vitro. When OD_600_ = 1, the *P. distasonis* were diluted to 10^−1^, 10^−2^, 10^−3^, 10^−4^, 10^−5^ times. Then, 5 μL diluent was dropped onto MRS Plates of 0 mM CdCl_2_, 1 mM CdCl_2_, 1 mM CdCl_2_ + 1 mM DHA, and 1 mM CdCl_2_ + 2 mM DHA, respectively, and incubated for 48 h [69].

### 4.11. Determination of Succinic Acid Content

Mouse feces were collected from each group (listed in Table 1) in the 7-day experiment and ground evenly. Then, 0.1 g feces was dissolved in a tube containing 900 μL 0.01 mol/L PBS, shaken and mixed, and centrifuged at 3000 rpm/min at 4 °C for 15 min. The supernatant was collected and processed according to the instructions from the succinic acid ELISA assay kit. The absorbance was then measured at a wavelength of 450 nm using an automatic enzyme label instrument Varioskan Flash (SPY-11234, Thermo Fisher, Waltham, MA, USA).

### 4.12. Statistical Analysis

Excel 2019 software (Microsoft, Redmond, Washington, DC, USA) was used to process the experimental data, GraphPad Prism 9.0 (GraphPad, San Diego, CA, USA) was used for the significance analysis, and Adobe Photoshop 2021 software (Adobe Systems, San Jose, CA, USA) was used for mapping. *p* < 0.05 was considered a significant difference.

## 5. Conclusions

This study suggests that DHA may outperform EPA in chelating Cd, reducing Cd accumulation, and promoting Cd excretion. DHA can restore the abundance of *Parabacteroides* in Cd-exposed mice. *P. distasonis* significantly reduced Cd toxicity and restored Cd-induced organ damage in mice. Succinic acid, the potential reactive substrate of Cd and the major product of *P. distasonis*, was also shown to promote Cd excretion. Not coincidentally, DHA can increase the succinic acid content in the feces. The data we have obtained support the idea that DHA may promote Cd excretion by restoring the abundance of *Parabacteroides* (e.g., *P. distasonis*) and increasing the production of succinic acid.

## Figures and Tables

**Table 1:** Experimental groups of mice.

Group	Number of Animals	Cd Concentration in Drinking Water (μM)	Intragastric Solution (μmol/g/d)
Control	6	-	-
Cd	6	100	-
Cd+ DHA	6	100	40
Cd+ EPA	6	100	40

**Table 2:** Experimental design of Cd-exposed mice treated with *P. distasonis*.

Group	Number of Animals	Cd Water Concentration (μM)	Intraperitoneally Injected Cd Dose (μg/d)	*P. distasonis* by Intragastric Administration (CFU/d)
Control (DW)	6	-	-	-
Control (IP)	6	-	-	-
Cd (DW)	6	100	-	-
Cd (IP)	6	-	9	-
Cd (DW) + *P. distasonis*	6	100	-	1 × 10^9^
Cd (IP) + *P. distasonis*	6	-	9	1 × 10^9^

**Table 3:** Experimental design of Cd-exposed mice treated with succinic acid.

Group	Number of Animals	Cd Water Concentration (μM)	Intraperitoneally Injected Cd Dose (μg)	Intragastric Dosage of Succinic Acid (mmol/g/d)
Control (DW)	6	-	-	-
Control (IP)	6	-	-	-
Cd (DW)	6	100	-	-
Cd (DW) + Succinic Acid (L)	6	100	-	20
Cd (DW) + Succinic Acid (H)	6	100	-	40
Cd (IP)	6	-	9	-
Cd (IP) + Succinic Acid (L)	6	-	9	20
Cd (IP) + Succinic Acid (H)	6	-	9	40

## Data Availability

The data that support the findings of this study are available from the corresponding authors upon reasonable request.

## Sample Availability

Samples of the compounds of all the DHA and cadmium chloride are not available from the authors.
